# BN-PVDF/rGO-PVDF Laminate Nanocomposites for Energy Storage Applications

**DOI:** 10.3390/nano12244492

**Published:** 2022-12-19

**Authors:** Okikiola Ganiu Agbabiaka, Miracle Hope Adegun, Kit-Ying Chan, Heng Zhang, Xi Shen, Jang-Kyo Kim

**Affiliations:** 1 Department of Mechanical and Aerospace Engineering, Hong Kong University of Science and Technology, Hong Kong, China; 2 Department of Aeronautical and Aviation Engineering, Hong Kong Polytechnic University, Hong Kong, China; 3 School of Mechanical and Manufacturing Engineering, University of New South Wales, Sydney, NSW 2052, Australia

**Keywords:** boron nitride nanosheets (BNNS), reduced graphene oxides (rGO), multiple layer structure, dielectric composites, charge energy density

## Abstract

The increasing demand for high energy storage devices calls for concurrently enhanced dielectric constants and reduced dielectric losses of polymer dielectrics. In this work, we rationally design dielectric composites comprising aligned 2D nanofillers of reduced graphene oxide (rGO) and boron nitride nanosheets (BNNS) in a polyvinylidene fluoride (PVDF) matrix through a novel press-and-fold technique. Both nanofillers play different yet complementary roles: while rGO is designed to enhance the dielectric constant through charge accumulation at the interfaces with polymer, BNNS suppress the dielectric loss by preventing the mobility of free electrons. The microlaminate containing eight layers each of rGO/PVDF and BNNS/PVDF films exhibits remarkable dielectric performance with a dielectric constant of 147 and an ultralow dielectric loss of 0.075, due to the synergistic effect arising from the alternatingly electrically conductive and insulating films. Consequently, a maximum energy density of 3.5 J/cm^3^—about 18 times the bilayer composite counterpart—is realized. The high thermal conductivities of both nanofillers and their alignment endow the microlaminate with an excellent in-plane thermal conductivity of 6.53 Wm^−1^K^−1^, potentially useful for multifunctional applications. This work offers a simple but effective approach to fabricating a composite for high dielectric energy storage using two different 2D nanofillers.

## 1. Introduction

The growing interest in electrical energy storage devices requires rational design of polymer-based composites with concomitantly high dielectric constants (*k*) and low dielectric losses (tan δ) [1,2,3,4,5,6,7]. Polymer dielectrics have received much attention because of their light weight, low cost, ease of processing, high breakdown strength ($E_{b}$), and higher flexibility than ceramic and metallic counterparts, making them suitable for emerging applications, such as wearable electronics, flexible energy storage devices, and hybrid vehicles [6]. Examples of polymers used for dielectrics include polyvinylidene fluoride (PVDF) [8], epoxy [9], polyimide (PI) [1], polyurethane (PU) [10], poly(ethylene terephthalate) (PET) [11], poly(methyl methacrylate) (PMMA) [12], and biaxially oriented polypropylene (BOPP) [13]. However, their low dielectric constants—usually below 10—greatly limit their practical applications [14].

Many attempts have been directed towards enhancing the *k* of polymer dielectrics, especially by incorporating high *k* ceramic fillers such as lead zirconate titanate $(\mathrm{Pb}[{\mathrm{Zr}}_{x}{\mathrm{Ti}}_{1-x}]O_{3}\left(0\leq x\leq 1\right)$ [15], calcium copper titanate $\left({\mathrm{CaCu}}_{3}{\mathrm{Ti}}_{4}O_{12}\right)$ [3], and barium titanate $\left({\mathrm{BaTiO}}_{3}\right)$ [16], into the polymer matrix. However, the improved *k* often comes at the expense of higher tan δ due to energy dissipation and high-field hysteresis losses. The mechanical properties of the composites also deteriorate because the high filler loading required to achieve high *k* tends to agglomerate and weaken the filler–matrix interfacial bond [10,17,18]. Meanwhile, the use of conductive fillers such as carbon nanotubes (CNTs) and graphene in a polymer matrix is a proven means of achieving high *k* ranging from 100 to 10,000 (at 1 kHz) at a low filler loading, thanks to large interfacial polarization aided by the wide contrast in electrical conductivity (EC) between the filler and the polymer matrix [19,20]. However, the tan δ values of the composites are unsatisfactory due to excessive current leakage arising from the formation of conductive pathways after reaching the percolation [21], prompting the need of solutions for suppressed leakage currents.

The use of electrically insulating two-dimensional (2D) materials such as boron nitride nanosheets (BNNS) as filler in polymer composites can prevent the leakage currents from developing by electrical conduction to lower the tan δ and improve the $E_{b}$ [1,10,20,22]. Compared to the bulk form of hexagonal boron nitride (h-BN), BNNS of large size (≥2 μm) with a mono or few-atomic-layer thickness are a preferred choice because of their high $E_{b}$ of ~800 MVm^−1^ [23], excellent thermal conductivity (TC) of over 360 Wm^−1^K^−1^ [24], and high-temperature stability [25]. However, using BNNS alone cannot satisfy the requirement of high *k* values due to their relatively low *k* below 4 [22], and indeed, the reported dielectric performance of BNNS composites is only unsatisfactory [26]. Moreover, to achieve TCs of composites ranging from 1 to 10 Wm^−1^K^−1^, a high BNNS content of over 30 wt.% is usually required, compromising the dielectric performance of composites for practical applications [27]. In fact, such high TCs could easily be realized with less than 10 wt.% graphene fillers that possess larger aspect ratios and much higher TCs (>2000 Wm^−1^K^−1^) than BNNS [28]. In addition, the inclusion of graphene can give rise to a drastically improved *k* of composites. However, the concurrent increase in electrical conductivity (EC) means an undesirable surge in tan δ, unable to employ polymer composites containing graphene fillers for dielectric applications [29]. Thus, designing electrically insulating composites with high *k* and low tan δ is very challenging yet highly desirable for achieving high-performance dielectrics, for example, by taking advantage of electrically insulating BNNS and electrically conducting graphene as hybrid fillers in polymer composites.

With the foregoing backdrop in mind, this work reports the development of composites consisting of PVDF as matrix, and reduced graphene oxide (rGO) and BNNS as reinforcing fillers that can deliver excellent dielectric performance [30]. PVDF is chosen for its $E_{b}$ and a relatively high *k* combined with a high energy density compared to other dielectric polymers [14]. The addition of rGO is designed to enhance the *k* through charge accumulation at the interfaces [19], while BNNS help suppress the tan δ by preventing the mobility of free electrons along the conduction pathways [1]. Both nanofillers possess excellent TCs [31]. The microlaminate composites designed in this work consist of alternating rGO/PVDF and BNNS/PVDF layers such that both high *k* and low tan δ values are achieved along with high $E_{b}$, charge energy densities, and TCs. The effects of number of alternating layers of electrically conducting and insulating composite films on dielectric performance are specifically studied.

## 2. Materials and Methods

### 2.1. Synthesis of rGO and BNNS

Graphene oxide (GO) was prepared using the modified Hummers’ method established previously [32], which is particularly suitable for synthesizing large GO sheets from natural graphite (supplied by Asbury Graphite Mills, Asbury, NJ, USA). In brief, 3 g of expanded graphite (EG) powder was dispersed in $H_{2}{\mathrm{SO}}_{4}$ (>95%, Fisher Scientific, Waltham, MA, USA) and $H_{3}{\mathrm{PO}}_{4}$ (85%, VWR Chemicals, Radnor, PA, USA) at a ratio of 360 to 40 mL. Then, 18 g of KMn$O_{4}$ (≥99%, Fluka, St. Gallen, Switzerland) was slowly added to the mixture, followed by magnetic stirring at 40 °C for 5 h. Afterward, the temperature was raised to 50 °C and the mixture was stirred for another 12 h before cooling down to room temperature. Next, 400 mL ice of deionized (DI) water was poured into the mixture, followed by adding 5 mL $H_{2}O_{2}$ (30%, Honeywell, Charlotte, NC, USA), yielding yellowish-brown GO suspension. The GO suspension was washed with 10% dilute HCl solution (37%, VWR Chemicals) on a centrifuge (Z326) at 12,000 rpm for 30 min which was repeated three times, followed by washing with DI water using the same centrifugation condition for three rounds. The collected GO was then reduced using hydrazine solution ($N_{2}H_{4}$, 35 wt.% in $H_{2}O$, Sigma-Aldrich, St. Louis, MI, USA). Next, 0.5 mL GO was added to 5 mL $H_{2}O$ and 5 μL $N_{2}H_{4}$ in a vial and stirred at 95 °C for 1 h. Then, 35 μL ${\mathrm{NH}}_{4}\mathrm{OH}$ (Uni-chem) was added to maintain the pH of the mixture at around 9–10 during the reduction process. A black precipitate was obtained, which was subsequently dried (Scientz-10N) at −50 °C for 24 h to yield rGO sheets.

Bulk h-BN powders (99.5%, 325 mesh, supplied by Alfa Aesar, Haffrey, MA, USA) were exfoliated to form a few-layer BNNS using a planetary ball-mill (DECO, 1617003) [10] at an h-BN to urea (≥99.0%, ACS, Washington, DC, USA) weight ratio of 1:30, operating at 400 rpm for 36 h. The urea had the following important functions: (i) aiding exfoliation of BNNS from the bulk h-BN; (ii) lubricating the milling balls to prevent BNNS from excessive mechanical damage; and (iii) functionalizing the BNNS to improve its dispersibility in polymer and solvent [10,33]. The milled product was diluted in DI water and washed several times by centrifugation, initially at 1000 rpm for 30 min to remove un-exfoliated thick h-BN sheets followed by washing of the supernatant at 12,000 rpm. The resultant BNNS were freeze-dried and collected for characterization and composite fabrication.

### 2.2. Fabrication of rGO/BNNS-PVDF Microlaminate Composites

**Figure 1:**
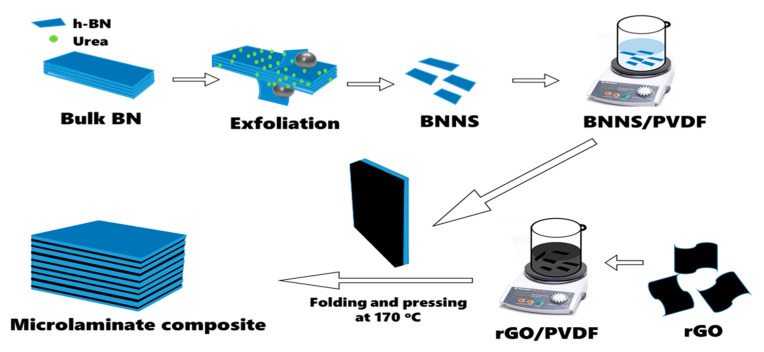
Schematic of BNNS exfoliation and fabrication procedure of microlaminate composites.

To prepare hybrid composites, films consisting of individual fillers (BNNS/PVDF and rGO/PVDF) were first prepared, followed by a hot pressing and folding process [34]. PVDF (Sigma-Aldrich, Mw~534,000 GPC powder) was dissolved in *N,N*-dimethylformamide (DMF, GC-99.8%, RCL Labscan, Bangkok, Thailand) solvent by magnetic stirring at 40 °C until a clear solution was formed. Then, the as-prepared BNNS fillers of varied weight fractions ranging from 10 to 30 wt.% were dispersed in DMF, which was mixed with PVDF solution by ultrasonication and stirring for 4 h to form a stable suspension. The suspension was cast onto a glass slide mold and dried in a vacuum oven (Memmert, 0173681). The as-prepared BNNS/PVDF films were collected from the mold after evaporating the solvent at 80 °C for 3 h. The BNNS/PVDF composite films are denoted as BN10, BN20, and BN30 according to their weight fractions. Similarly, rGO/PVDF films were prepared by dispersing rGO particles of different weight fractions ranging from 1 to 10 wt.% in DMF before mixing with PVDF solution. The rGO/PVDF composite films are denoted as rGO1, rGO3, rGO5, and rGO10 according to the weight fractions of rGO. Bilayer hybrid filler composites were prepared by stacking each of the BNNS/PVDF and rGO/PVDF composite films and pressing at 170 °C and ~3 MPa, which are designated as G/BNXX where XX stands for the weight fraction of BNNS in the BNNS/PVDF film. Here, G refers to the 10 wt.% rGO/PVDF composite which was selected for further study after preliminary experiments. Thereafter, the bilayer composites were subjected to folding and pressing at 170 °C and 3 MPa to form hybrid microlaminate composites of G/BNXX_YY with different total numbers of layers, where YY = 4, 8, 16, and 32 layers. A schematic of BNNS exfoliation and fabrication of hybrid microlaminate composites is shown in Figure 1, while the details of their designations and the corresponding filler weight fractions, number of layers, and thicknesses are presented in Table S1.

### 2.3. Characterization

The morphologies of h-BN, BNNS, and the cryo-fractured surface of the composites were characterized on a scanning electron microscope (SEM, JEOL JSM 6390) at an acceleration voltage of 20 kV. Raman spectroscopy (Reinshaw microRaman) was used to examine h-BN before and after exfoliation. The bonding characteristics and functional groups on BNNS before and after exfoliation were examined using the FTIR spectroscopy (Bruker Vertex 70Hyperion 1000) at wavelengths ranging from 400 cm^−1^ to 4000 cm^−1^. The crystallization of bulk h-BN and BNNS was studied on an X’pert Pro (PANalytical) diffractometer with Cu Kα (λ = 0.154 nm) radiation. The dielectric properties of the composites and neat PVDF were measured at room temperature using a precision LCR meter (E4980A) in the frequency range from 100 Hz to 1 MHz according to the specification, ASTM D150. The breakdown voltage of composites was measured using a Gamma high-voltage (HV) power supply (Model No-RR20-30R/220). Briefly, an electrode of 1 cm × 1 cm in size was connected to the HV power source and placed in contact with both sides of the samples. Afterwards, the voltage was increased in a stepwise manner until the samples were mechanically failed. The breakdown strength was calculated by dividing the breakdown voltage by the sample thickness. The TC of composites was measured using a TPS 2500S hot disk. Three samples were tested for each set of conditions for the dielectric property, breakdown voltage, and TC measurements.

## 3. Results and Discussion

### 3.1. Exfoliation of BNNS

The exfoliation of h-BN is challenging due to the strong lip–lip interactions between its interlayers [35]. However, several methods have been explored to exfoliate h-BN into ultrathin BNNS. Among them, ball milling has proven to be effective, less time-consuming, giving rise to the highest yield of BNNS [33,36], making the technique attractive for adoption in this study. To minimize potential structural damage to BNNS due to high-speed ball collision during milling, a relatively low milling speed of 400 rpm was chosen, and urea [10] was added in the ball milling chamber to serve as intercalant and lubricant during exfoliation. The urea molecules helped reduce the effect of ball collision by adsorbing onto the h-BN surfaces and enlarged the interlayer spacing by intercalation of h-BN layers for easy exfoliation of BNNS. Figure S1a,b present bulk h-BN platelets of large thicknesses and varying lateral sizes and BNNS obtained after exfoliation, respectively. The AFM image and the corresponding height profile given in Figure S1c,d indicate that the BNNS had a lateral size of ~2 μm and a thickness ranging from 7 to 10 nm. This implies that the impact of ball collision was mild and did not cause severe fragmentation of h-BN into smaller pieces during exfoliation. The XRD patterns shown in Figure S1e exhibit a prominent (002) peak at 2θ = 26.93° with a d-spacing of 3.308 Å for h-BN. After exfoliation, its intensity was significantly reduced and the peak position was downshifted to 2θ = 26.79° giving rise to a larger d-spacing of 3.324 Å, which is consistent with previous findings [1]. In addition, the intensities of other minor diffraction peaks of the (100), (101), (102), (004), (104), and (110) planes were reduced after exfoliation, while no urea peak was identified. The intensity ratio of the (100) to (004) plane peaks, I_(100)_/I_(004)_, decreased from 0.64 to 0.30 after exfoliation, which signifies the alignment of BNNS in the horizontal plane as a result of their high aspect ratios [1,37]. The thickness of BNNS on the crystalline plane (002) at 26.79° was calculated to be 9.93 nm according to the Debey–Scherrer equation: t=KλFWHM.cosθ, where FWHM is the full width at half maximum, θ is the peak position, λ is the wavelength of X-ray, and K is the shape factor. This value falls within the thickness range measured from the AFM findings.

The Raman spectra of h-BN and BNNS shown in Figure S1f exhibit noticeable G-band peaks at 1365 cm^−1^ and 1366 cm^−1^, respectively, corresponding to the E2g mode vibration. The marginal upshift of the peak after exfoliation indicates weaker interlayer interactions in BNNS, which corroborates the enlarged d-spacing [1]. There was significant broadening of the G-band peak of BNNS after exfoliation with an increase in FWHM from 11.7 to 13.0, which is attributed to stronger surface scattering influencing the vibrational excitation [35,38]. Their chemistry and bonding characteristics were analyzed using the FTIR spectra. Both materials exhibited similar bond characteristics at ~759 and ~1375 cm^−1^, as shown in Figure S1g, corresponding to the B-N in-plane bending and B-N out-of-plane stretching vibrations, respectively. However, additional broad band peaks appeared at 3221 and 3406 cm^−1^ for BNNS, corresponding to the N-H stretching and O-H stretching, respectively. The former indicates the functionalization of BNNS by urea, while the latter reflects the effect of centrifugal washing after ball milling.

### 3.2. Structure and Dielectric Properties of Microlaminate Composites

To understand the contributions from the individual fillers to the dielectric performance of hybrid filler composites, laminates were fabricated by initially stacking two films with different filler types followed by folding and pressing. The folding technique was used simply because it is more convenient than the direct stacking approach and there is no need to cut the composites into pieces at high temperature before stacking. In addition, the technique is effective in controlling the distribution of fillers and builds up multiple alternating layers in the microlaminate composites so that an optimal structural configuration can be identified. Typically, composites with high 2D nanofiller loadings often encounter difficulties of uniform dispersion due to the high tendency of agglomeration of these fillers, resulting in severely deteriorated mechanical, physical, and dielectric performance of the composites.

**Figure 2:**
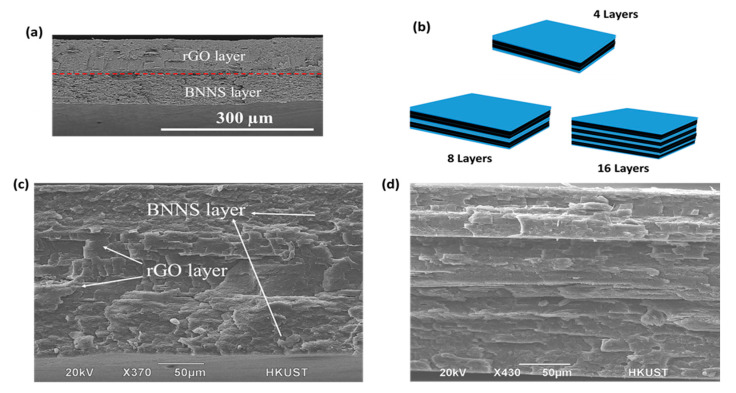
(**a**) SEM image of cryo-fractured edge surface of G/B30 bilayer composite. (**b**) Schematics of 4, 8, and 16-layer microlaminate composites; the blue and black layers represent the BNNS/PVDF and rGO/PVDF composite films, respectively. (**c**,**d**) SEM image of cryo-fractured edge surface of respective 4 and 32-layer microlaminate composites.

The folding and pressing process was carried out at 170 °C below the melting point of PVDF after trial-and-error experiments to avoid both complete fusion of the layers and thus disruption of filler orientation in the in-plane direction. Figure 2a presents the SEM image of G/B30 bilayer composite consisting of an electrically insulating BNNS/PVDF layer firmly bonded together with an electrically conducting rGO/PVDF layer. The composite of four layers (G/B30_4L) was prepared by folding the G/B30 film such that the BNNS/PVDF films formed the outer layers, while the two layers of rGO/PVDF film were stacked as inner layers. The same process was repeated until the 32-layer microlaminate composite containing 16 layers each of rGO/PVDF and BNNS/PVDF films was obtained, as schematically shown in Figure 2b. It is worth mentioning that the hot-pressing process made the nanofiller distribution within each layer highly aligned, as shown in Figure 2c,d.

**Figure 3:**
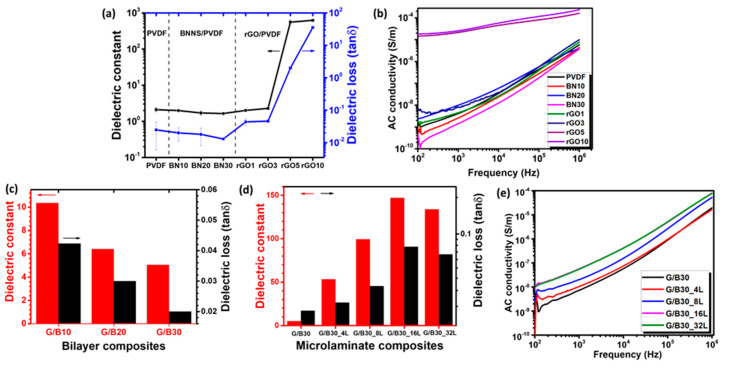
(**a**) Dielectric constant and loss at 1 kHz and (**b**) AC conductivity of PVDF, rGO/PVDF, and BNNS/PVDF films. (**c**) Dielectric constant and loss at 1 kHz of bilayer composites. (**d**) Dielectric constant and loss at 1 kHz and (**e**) AC conductivity of microlaminate composites comprising different numbers of rGO/PVDF and BNNS/PVDF films.

In the preliminary experiments, the effects of individual fillers on dielectric properties and electrical conductivities of the composites were investigated. The *k* and tan δ curves of neat PVDF, BNNS/PVDF, and rGO/PVDF single-layer composite films and G/B bilayer composites plotted as a function of frequency are shown in Figure S2, and the *k* and tan δ values measured at a frequency of 1 kHz are summarized in Figure 3a,c,d. As shown in Figure 3a, both the *k* and tan δ of BNNS/PVDF composites gradually decreased as the BNNS wt.% increased, even below the neat PVDF counterparts, due to the insulating nature of BNNS fillers in the PVDF matrix. Conversely, the *k* of rGO/PVDF composites drastically increased after the incorporation of rGO fillers of above 5 wt.% and reached the highest value of 628 at 10 wt.% due to the formation of percolating network by the highly conducting rGO sheets. This finding is consistent with the electrical conductivity shown in Figure 3b where the composites with rGO filler content higher than 5 wt.% behaved like a conductor. The Maxwell–Wagner–Sillars (MWS) effect explains the interfacial polarization, which was induced by charge entrapment in the composite because of the inclusion of conducting rGO sheets. Despite showing a great potential for high energy storage capacities due to their high *k* values [19], the rGO/PVDF composites alone cannot be used for meaningful applications because of their high tan δ values, far above the practically viable level of 0.1 [10].

In an attempt to simultaneously achieve high *k* and low tan δ values, hybrid composites were fabricated by alternatingly stacking conducting rGO/PVDF and insulating BNNS/PVDF films. Initially, a single layer of the BNNS/PVDF composite containing varying BNNS contents was stacked on top of a single layer of the rGO10 composite to form bilayer composites, designated as G/B10, G/B20, and G/B30. The rGO10 composite was selected to ensure that the rGO filler content of bilayer composites was 5 wt.%. The dielectric properties of bilayer composites are shown in Figure S2 and Figure 3c. While their tan δ values were sufficiently low, the absolute *k* values ranged from 5.1 to 10.4, much enhanced compared to the neat PVDF or all BNNS/PVDF and rGO1, rGO3 composite counterparts, due to the synergy arising from the presence of both the electrically insulating and conducting layers. It is also worth noting that both the *k* and tan δ deteriorated with increasing BNNS content, indicating that the BNNS dominated the dielectric performance of bilayer composites such as the single-layer BNNS/PVDF film (Figure 3a). Given the lowest tan δ of 0.02, the G/B30 bilayer composite was chosen to fabricate alternating multilayer structures, and their dielectric properties are plotted as a function of frequency in Figure S3 and the results are summarized in Figure 3d. Interestingly, the *k* of the multilayer microlaminate composites increased significantly as the number of rGO/PVDF layers increased with a simultaneous increase in tan δ, but within the acceptable range below 0.1 [10]. The *k* augmented from ~5.1 of the bilayer composite (G/B30) to ~147 of the 16-layer microlaminate (G/B30_16L), whereas the corresponding tan δ increased to ~0.08 when measured at 1 kHz. Due to the large differences in conductivity and dielectric performance between the rGO/PVDF and BNNS/PVDF films, the effect of MWS interfacial polarization became more conspicuous when increasing the number of layers, which was mainly responsible for the surge in dielectric constant. The result also shows that the 32-layer microlaminate composite displayed a slightly lower *k* of ~140 than the 16-layer microlaminate counterpart and a marginally lower tan δ of 0.07. This observation is probably associated with the sliding caused by composite layer infusion at a higher number of layers during the hot press process [39,40]. It also implies that the microlaminate composite consisting of numerous micro-capacitors of rGO/PVDF and BNNS/PVDF layers became affected by the inhomogeneous boundaries as a result of uneven loading, thereby degrading the dielectric constant. The lower AC conductivities of the microlaminate composites than the rGO5 and rGO10 counterparts, as shown in Figure 3e, further suggest the generally insulating nature of the composites, which is ascribed to the multiple insulating BNNS/PVDF layers, suppressing the tan δ below 0.1.

**Figure 4:**
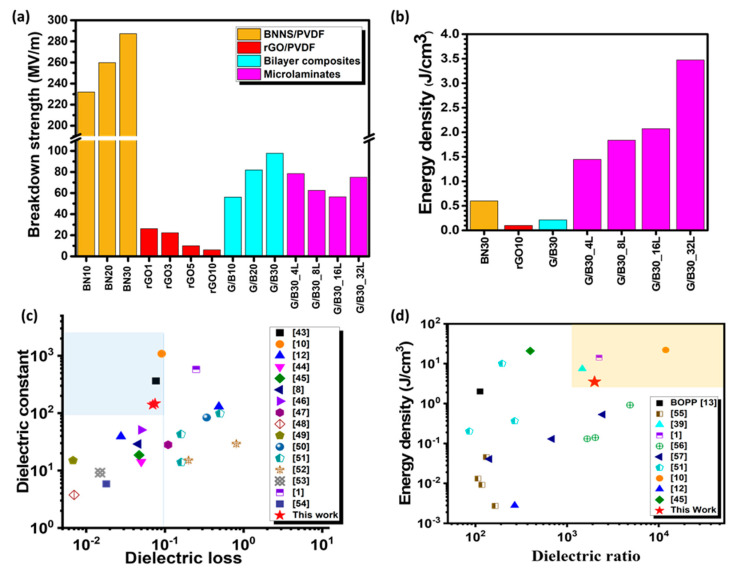
(**a**) Breakdown strength of rGO/PVDF, BNNS/PVDF, bilayer, and microlaminate composites. (**b**) Energy density of single-layer, bilayer, and microlaminate composites. (**c**) Comparison of dielectric constant versus dielectric loss and (**d**) energy density versus dielectric ratio among the current microlaminate composite and other rGO- and/or BNNS-based polymer composites reported in the literature. The light-blue region in (**c**) and light-orange region in (**d**) are considered ideal ranges of properties for dielectric composites.

Figure 4a shows the average breakdown strength $E_{b}\;$of the composites calculated according to the specification, ASTM Standard D149-09 [41]. The bilayer and microlaminate composites had excellent $E_{b}$ up to a maximum of 97.8 and 78.4 MV/m, respectively, about 16 and 13 times that of the rGO10 composite acting alone without BN30 insulating films. The inherently high breakdown strength of BN30 composites—about 287.2 MV/m—and its excellent insulating barrier characteristics among the rGO10 layers contributed to the enhanced breakdown strengths of the bilayer and microlaminate composites. As expected, with increasing the filler content, the $E_{b}$ of monolayer BNNS/PVDF composites increased, whereas that of monolayer rGO/PVDF composites decreased. A similar trend was observed for bilayer composites whereby the $E_{b}$ increased as the BNNS wt.% increased. However, among the microlaminates, the composite with four layers showed the highest $E_{b}$ value of 78.4 MV/m, which decreased as the number of layers increased to 56.4 MV/m for a 16-layer composite, except for a 32-layer composite, which showed a higher $E_{b}$ value of 75 MV/m. The multilayer laminates containing the same BNNS and rGO contents presented slightly lower $E_{b}$ than the bilayer counterpart. In this case, the $E_{b}$ was adversely affected by the thicker composite laminates than the bilayer composites. The observation also indicates that one thick film of strong BN30/PVDF in the bilayer composite was able to withstand the applied electric field better than the multiple BN30/PVDF films sandwiched by weak rGO10/PVDF films in the multilayer microlaminates. The dielectric breakdown mechanism is schematically presented in Figure S4, where the electrical tree or conduction pathway (indicated by the red arrows) in the polymer matrix was responsible for the composites to breakdown under an applied voltage field [42]. In other words, the hindrance of electrical tree growth by the insulating BNNS fillers as well as the layered structure translates to a better breakdown strength exhibited by the microlaminate composites when compared to monolayer rGO/PVDF counterparts.

The energy stored in a dielectric capacitor can be expressed in terms of electric field ($E_{f}$) and electric displacement (D) as $U_{e}$ = $\int E_{f}dD$. However, for linear dielectric materials whose dielectric constant is not dependent on the external electric field, the energy stored ($U_{e}$) can be further expressed as: $0.5\varepsilon_{0}kE_{b}{}^{2}$**,** where $\varepsilon_{0}$ (=$8.85\times 10^{-12}$ Fm^−1^) is the dielectric constant of vacuum. In Figure 4b, the energy density of the microlaminate composites improved with increasing the number of layers. The G/B30_32L composite had the highest value of 3.5 J/cm^3^, which is about 6 and 35 times those of the BN30 and rGO10 single-filler composites, respectively. The value is even higher than that of the conventional dielectric, biaxially oriented polypropylene (BOPP), for energy storage applications [13]. Although the microlaminate composite delivered a relatively lower dielectric constant than rGO10 and a lower breakdown strength than BN30, it had great energy storage potential compared to these single-filler composites acting alone. This finding emphasizes the importance of striking a balance between dielectric constant, dielectric loss, and breakdown strength to achieve an optimized energy density of composites. In summary, the high energy density of the microlaminate composites recorded in this work can be attributed to the synergy arising from the alternatingly layered architecture of conducting rGO/PVDF and insulating BNNS/PVDF films, which contributes to its relatively high dielectric constant and well-suppressed dielectric loss and high breakdown strength.

The dielectric performance and energy density of selected microlaminate composites are compared with those of the dielectric composites reported in the open literature as shown in Table 1 and Table 2 [1,8,10,12,39,43,44,45,46,47,48,49,50,51,52,53,54,55,56,57], and the summary comparisons are plotted in Figure 4c,d. Given the counteracting characteristics between dielectric constant and dielectric loss, the dielectric ratio—the ratio of dielectric constant to dielectric loss—is considered as a basis for comparison to measure the overall dielectric performance of the composites. It can be seen that the microlaminate composites reported in this work possess dielectric performance highly competitive with reported values or even better than the majority, indicating that the design approach and the method adopted for fabricating the microlaminate composites are sensibly demonstrated for high dielectric constants and suppressed dielectric losses for emerging energy storage applications.

### 3.3. Thermal Properties of the Composites

**Figure 5:**
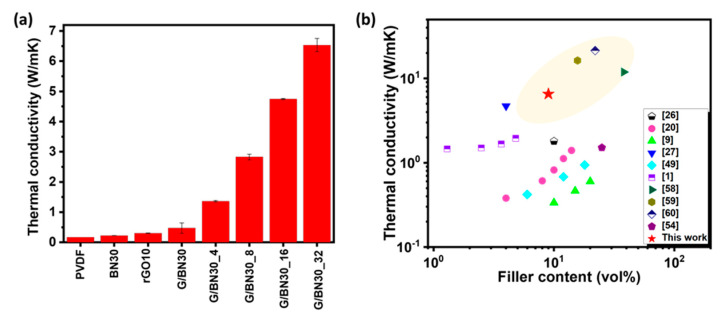
(**a**) Thermal conductivities of neat PVDF, single-layer, bilayer, and microlaminate composites. (**b**) Comparison of thermal conductivities among the current microlaminate composite and other rGO- and/or BNNS-based polymer composites reported in the literature.

Although the microlaminate composites showed a good dielectric performance, it is important to investigate their TC for multifunctional applications because dielectric materials must also withstand and effectively dissipate heat during service. While polymer-based dielectrics are attractive for miniaturizing electrical storage devices, their low TC values are often a major setback for practical applications [54]. It is not uncommon for dielectric materials to exhibit a conduction loss phenomenon where the dielectric loss is converted into heat during service. Unless the heat is properly removed, early breakdown of the material may take place [20]. Figure 5a shows the TC of selected single-layer and bilayer composites as well as microlaminate composites. It is shown that both the single-layer and bilayer composites showed an improved TC compared to the neat PVDF. The TC of the microlaminates increased almost linearly with increasing the number of constituent layers, reaching the highest value of 6.53 Wm^−1^K^−1^ for the 32-layer composite, which is equivalent to 38-, 27-, and 13-fold enhancement compared to the neat PVDF (0.17 Wm^−1^K^−1^), single-layer and bilayer composites, respectively. Aside from the inherently high TCs of rGO and BNNS, the remarkable TC enhancement, especially for the composites consisting of a large number of layers, is attributed to the better alignment of 2D fillers in the plane direction. Figure 5b and Table 3 [1,9,20,26,27,49,54,58,59,60] compare the TCs of the current microlaminate and state-of-the-art values reported for other rGO and BNNS polymer composites, presenting a relatively high TC value for the microlaminates when the filler content is taken into account.

## 4. Conclusions

This work demonstrates simultaneous achievement of high dielectric constants and ultralow dielectric losses of hybrid composites made by electrically conducting reduced graphene oxide (rGO) and electrically insulating boron nitride nanosheets (BNNS) fillers in a PVDF matrix via a folding and pressing process to form an alternating multilayer microlaminate composite. The microlaminate composite assembled with 16 layers each of rGO/PVDF and BNNS/PVDF films delivered a maximum dielectric constant of 140 and an ultralow dielectric loss of ~0.07 at 1 kHz, as well as a high energy density of 3.5 J/cm^3^. The remarkable dielectric performance of the composite was attributed to the synergy arising from the hybrid fillers of BNNS and rGO, as well as the alternating multilayer structure. The electrically conducting rGO/PVDF layers helped augment the dielectric constant, while the electrically insulating BNNS/PVDF layers suppressed the leakage currents to reduce the dielectric loss and improve the breakdown strength. Furthermore, the microlaminate composite exhibited an excellent thermal conductivity of 6.53 Wm^−1^K^−1^, arising from the highly thermally conducting fillers and their significant alignment during the pressing and folding process, thereby allowing efficient heat transport along the in-plane direction. The rational design of layer-structured laminates through the folding and pressing approach developed in this work will offer new insights into the achievement of high dielectric performance for high energy storage applications of rGO and BNNS hybrid polymer composites.

## Figures and Tables

**Table 1:** Comparison of dielectric constants and losses of our microlaminate composites with other composites reported in the literature.

Materials	At 1 kHz		Ref.
	Dielectric Constant	Dielectric Loss (tan δ)	
BaTiO3/Graphite/PDMS	73.5	0.19	[43]
rGO-PU/BN-PU	1084	0.091	[10]
BT@HBP/PMMA-4	130	0.485	[12]
BT@HBP@PMMA-4	39.3	0.0276	
PVDF/Mxene@CTAB	82.1	0.2	[44]
PVDF/P(VDF-TrFE-CTFE)/PVDF	18.61	0.047	[45]
Mxene/PVDF	29	0.046	[8]
rGO-TiO_2_/PVDF	211	0.104	[46]
PVDF/hBN/SiC	28.1	0.11	[47]
mBN/PI	3.77	0.007	[48]
epoxy/h-BN/rGO	15	0.0068	[49]
SMG/PVDF	83.8	0.34	[50]
GROH/PVDF	98.91	0.501	[51]
	42.9	0.16	
	13.91	0.161	
FGS/PP	29.4	0.81	[52]
	15.2	0.2	
Ag-PDOP/PVDF	9.2	0.015	[53]
rGO-PI/BNNS-PI	579	0.25	[1]
BN/CNT/PVDF	5.86	0.018	[54]
G/B30_16L	147.1	0.075	This work
G/B30_32L	139.5	0.07	

PDMS: polydimethylsiloxane; HBP: hyperbranched aromatic polyamide; BT: barium titanate; GROH: hydroxylated graphene; CTAB: cetyltrimethylammonium bromide; P(VDF-TrFE-CTFE: poly(vinylidenefluoride-ter-trifluoroethylene-ter-chlorotrifluoroethylene); SMG: surface-modified graphene; FGS: functionalized graphene sheets; PP: polypropylene; Ag-PDOP: silver-polydopamine.

**Table 2:** Comparison of energy density as a function of dielectric ratio of our microlaminate composites with other composites reported in the literature.

Materials	Dielectric Ratio	Energy Density (J/cm^3^)	Ref.
GO/CNT/PU	164	0.0027	[55]
	117	0.0092	
	107	0.013	
	131	0.046	
PPD-CFGO/PI	1656	0.13	[56]
	2030	0.14	
	4857	0.91	
Dopamine@BCZT/PVDF	143	0.041	[57]
	684	0.13	
	2446	0.53	
GROH/PVDF	86	0.20	[51]
	268	0.37	
	196	10.07	
rGO-PU/BN-PU	11912	22.70	[10]
Mxene/PVDF	1464	7.40	[39]
BT@HBP/PMMA-4	268	0.0028	[12]
PVDF/P(VDF-TrFE-CTFE)/PVDF	396	20.86	[45]
rGO-PI/BNNS-PI	2316	14.20	[1]
G/B30_32L	1993	3.50	This work

PPD: *p*-Phenylenediamine; CFGO: carboxyl-functionalized GO; BCZT: calcium barium zirconate titanate (Ba_0.95_Ca_0.05_Zr_0.15_Ti_0.85_O_3_).

**Table 3:** Comparison of thermal conductivities of our microlaminate with other rGO and/or BNNS polymer composites reported in the literature.

Materials	Filler Content (Vol%)	Thermal Conductivity (Wm^−1^K^−1^)	Ref.
c-BCB/BNNS	10	1.80	[26]
GO/BNNS/PVA	38.01	11.90	[58]
BNNS/P(VDF-TrFE-CFE)	14	1.40	[20]
	8	0.61	
BNNS/epoxy	20	0.60	[9]
	10	0.34	
BNNS/PVDF	15.71	16.30	[59]
BNNS/PVA	22	21.40	[60]
BNNS/PVDF	4	4.69	[27]
BNNS/rGO/epoxy	18	0.94	[49]
rGO-PI/BNNS-PI	1.30	1.49	[1]
	4.82	1.95	
m-h-BN/MWCNTs-SiO_2_/PVDF	25	1.51	[54]
G/B30_32L	10	6.53	This work

c-BCB: crosslinked divinyltetramethyldisiloxane-bis(benzocyclobutene); PVA: polyvinyl alcohol; MWCNTs: multi-walled carbon nanotubes.

## Data Availability

Not applicable.

## Supplementary Materials

The following supporting information can be downloaded at: https://www.mdpi.com/article/10.3390/nano12244492/s1, Figure S1: Morphology, structure, and chemistry of bulk BN and BNNSs. (a,b) SEM images, (c,d) AFM image and the corresponding height profile of BNNS. (e) XRD patterns, (f) Raman spectra and (g) FTIR spectra of bulk BN and BNNS; Figure S2: (a,c,e) Dielectric constant (*k*) and (b,d,f) dielectric loss (tan δ) of neat PVDF, BNNS/PVDF, and rGO/PVDF single-layer composite films and G/B bilayer composites as a function of frequency ranging from 10^2^ to 10^6^ Hz; Figure S3: (a) Dielectric constant (*k*) and (b) dielectric loss (tan δ) of microlaminate composites with different number of layers as a function of frequency ranging from 10^2^ to 10^6^ Hz; Figure S4: Schematic of electrical pathways in composites; Table S1: Designations of composite materials prepared in this work.
