# Supplementary material for: BN-PVDF/rGO-PVDF Laminate Nanocomposites for Energy Storage Applications
Source: Nanomaterials (Basel). 2022 Dec 19;12(24):4492. doi: 10.3390/nano12244492 (PMC9781690; doi:10.3390/nano12244492)
Supplement: Supplementary file 1 [file nanomaterials-12-04492-s001.zip › nanomaterials-2075454-supplementary.pdf]

Supporting Information

BN-PVDF/rGO-PVDF laminate Composites for Energy Storage Applications

Okikiola Ganiu Agbabiaka[1], Miracle Hope Adegun[1], Kit-Ying Chan[1,2], Heng Zhang[1], Xi Shen[1,2] and Jang-Kyo Kim[1,3*]

[1]Department of Mechanical and Aerospace Engineering, The Hong Kong University of Science and Technology, Clear Water Bay, Kowloon, Hong Kong

[2]Department of Aeronautical and Aviation Engineering, The Hong Kong Polytechnic University, Hung Hom, Kowloon, Hong Kong

[3]School of Mechanical and Manufacturing Engineering, The University of New South Wales,

Sydney, NSW 2052, Australia

*Corresponding email: mejkkim@ust.hk

Table S1. Designations of composite materials prepared in this work.

Materials Designations		Filler weight fraction (%)		No. of layers	Thickness (μm)
		BNNS	rGO		
BNNS/PVDF films	BN10	10	-	-	90
	BN20	20	-	-	90
	BN30	30	-	-	90
rGO/PVDF films	rGO1	-	1	-	90
	rGO3	-	3	-	90
	rGO5	-	5	-	90
	rGO10	-	10	-	90
Bilayer composites (G = 10 wt% rGO/PVDF; B = BNNS/PVDF)	G/B10	5	5	-	180
	G/B20	10	5	-	180
	G/B30	15	5	-	180
Microlaminate composites	G/B30_4L	15	5	4	240
	G/B30_8L	15	5	8	300
	G/B30_16L	15	5	16	330
	G/B30_32L	15	5	32	300

The densities of BNNS (ρ_{BNNS}) and rGO (ρ_{rGO}) are 2.1 g/cm^3 and 1.91 g/cm^3, respectively.

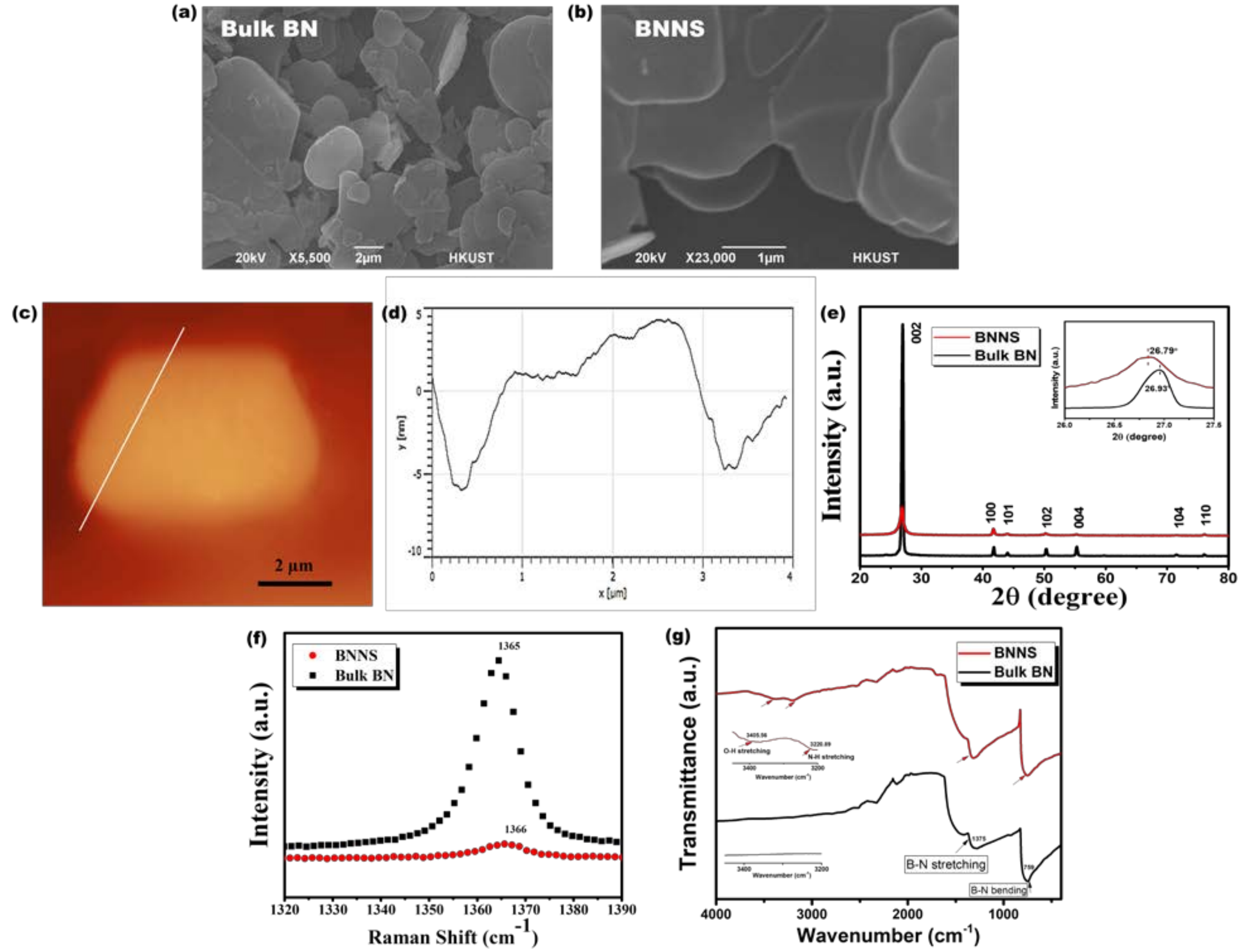

Figure S1. Morphology, structure and chemistry of bulk BN and BNNSs. (a-b) SEM images, (c-d) AFM image and the corresponding height profile of BNNS. (e) XRD patterns, (f) Raman spectra and (g) FTIR spectra of Bulk BN and BNNS.

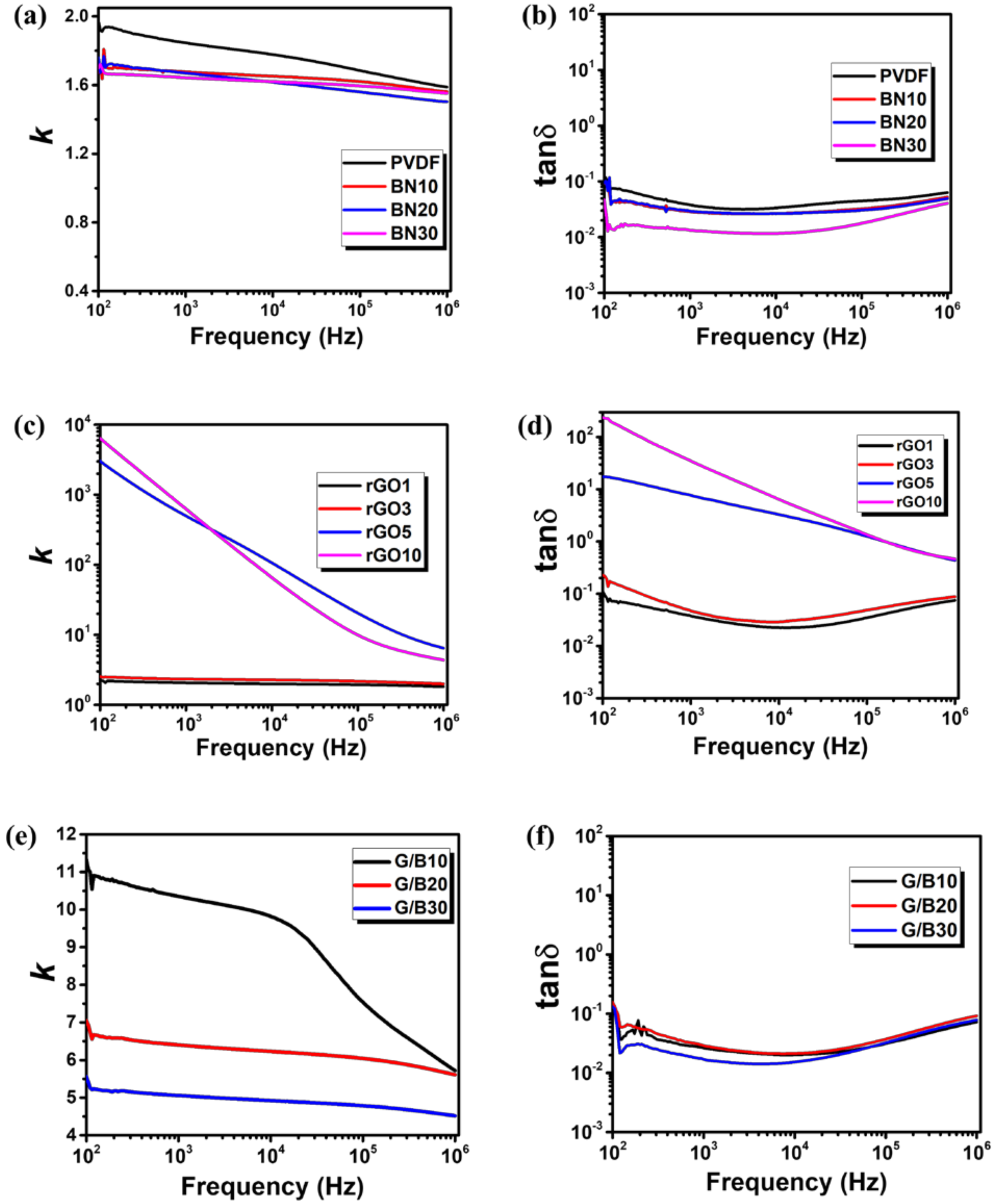

Figure S2. (a, c, e) Dielectric constant (*k*) and (b, d, f) dielectric loss (*tan δ*) of neat PVDF, BNNS/PVDF and rGO/PVDF single-layer composite films and G/B bilayer composites as a function of frequency ranging 10^2-10^6 Hz.

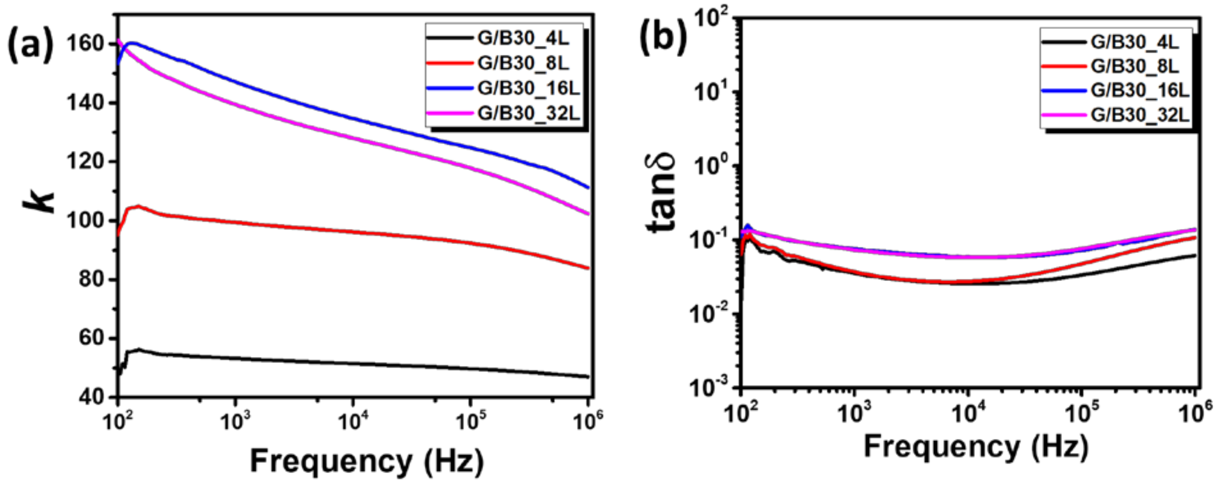

Figure S3. (a) Dielectric constant (*k*) and (b) dielectric loss (*tan δ*) of microlaminate composites with different number of layers as a function of frequency ranging 10^2-10^6 Hz.

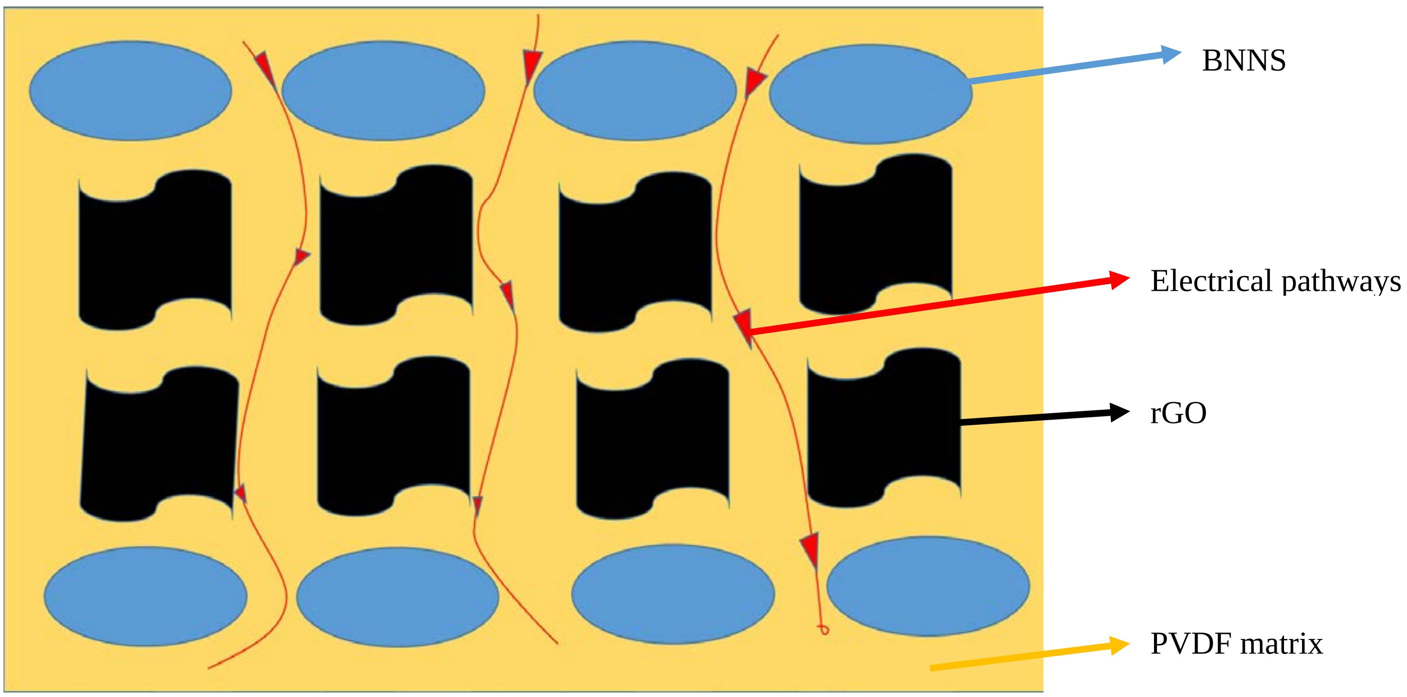

Figure S4. Schematic of electrical pathways in composites.
